# A direct comparison of natural and acoustic-radiation-force-induced cardiac mechanical waves

**DOI:** 10.1038/s41598-020-75401-1

**Published:** 2020-10-28

**Authors:** Lana B. H. Keijzer, Annette Caenen, Jason Voorneveld, Mihai Strachinaru, Daniel J. Bowen, Jens van de Wouw, Oana Sorop, Daphne Merkus, Dirk J. Duncker, Antonius F. W. van der Steen, Nico de Jong, Johan G. Bosch, Hendrik J. Vos

**Affiliations:** 1 Department of Cardiology, Erasmus MC, Rotterdam, The Netherlands; 2 IBiTech-bioMMeda, Ghent University, Ghent, Belgium; 3 Cardiovascular Imaging and Dynamics Lab, Catholic University of Leuven, Leuven, Belgium; 4 Department of Imaging Physics, Delft University of Technology, Delft, The Netherlands

**Keywords:** Cardiology, Acoustics, Imaging techniques, Echocardiography

## Abstract

Natural and active shear wave elastography (SWE) are potential ultrasound-based techniques to non-invasively assess myocardial stiffness, which could improve current diagnosis of heart failure. This study aims to bridge the knowledge gap between both techniques and discuss their respective impacts on cardiac stiffness evaluation. We recorded the mechanical waves occurring after aortic and mitral valve closure (AVC, MVC) and those induced by acoustic radiation force throughout the cardiac cycle in four pigs after sternotomy. Natural SWE showed a higher feasibility than active SWE, which is an advantage for clinical application. Median propagation speeds of 2.5–4.0 m/s and 1.6–4.0 m/s were obtained after AVC and MVC, whereas ARF-based median speeds of 0.9–1.2 m/s and 2.1–3.8 m/s were reported for diastole and systole, respectively. The different wave characteristics in both methods, such as the frequency content, complicate the direct comparison of waves. Nevertheless, a good match was found in propagation speeds between natural and active SWE at the moment of valve closure, and the natural waves showed higher propagation speeds than in diastole. Furthermore, the results demonstrated that the natural waves occur in between diastole and systole identified with active SWE, and thus represent a myocardial stiffness in between relaxation and contraction.

## Introduction

Heart failure affects 1–2% of the adult population in developed countries^1^, and its social burden in terms of mortality, morbidity and healthcare costs is increasing due to the aging population^2–4^. Heart failure is a progressive chronic condition, typically preceded by cardiac remodeling—defined as structural and functional changes of the heart in response to altered hemodynamic loading and/or cardiac injury^5^. Current assessment of cardiac remodeling is incomplete as it relies on the ultrasonic measurement of cardiac volume, flow and tissue velocity; but not on the intrinsic mechanical properties of the heart^6^. Thus, a non-invasive technique that directly measures cardiac stiffness properties in vivo is highly desirable, since it could provide better understanding of the pathophysiology of heart failure. Such a direct assessment of tissue properties could be of particular value in the diagnosis of heart failure with preserved ejection fraction, in which diastolic dysfunction caused by an increase in myocardial stiffness, predominates. Furthermore, such a technique could serve as a screening tool for cardiac remodeling and be useful to stratify patients in order to accommodate personalized treatment^1^.

Natural and active ultrasound-based shear wave elastography (SWE) are two promising methods that can potentially measure tissue elasticity. Natural and active SWE methods study the speed of the vibrations propagating over the myocardium, often referred to as shear waves or SWs. In theory, higher propagation speeds are expected for stiffer materials, resulting in a quantitative measure of tissue stiffness. Natural SWE analyzes the natural transverse vibrations induced in the myocardium after aortic and mitral valve closure (AVC and MVC)^7–13^, whereas active SWE uses a focused high-energy ultrasonic beam generating an acoustic radiation force (ARF) to induce a perturbation in the tissue of interest^14–18^. In this study, we will focus on the relationship between the natural vibrations after AVC and MVC and active SWE.

There is an increasing number of studies demonstrating the feasibility of SWE for the characterization of cardiac stiffness, of which many were performed in a research context: (i) natural SWE in in vivo animals^19^ and in vivo healthy volunteers^12,13^; and (ii) active SWE in ex vivo animals^20–22^, ex vivo Langendorff-perfused animals^23–25^, in vivo open-chest animals^14,15,24,26^ and in vivo healthy volunteers^18^. It is only very recently that various clinical studies have shown the potential of natural and active SWE to distinguish SW properties of the myocardium in different patient groups from healthy volunteers. Higher propagation speeds were measured in hypertrophic cardiomyopathy patients than in healthy volunteers, using natural SWs after AVC^8^ and actively induced SWs at end-diastole^27^. Also, higher propagation speeds were found in cardiac amyloidosis patients when analyzing natural SWs after AVC and MVC^9^. Furthermore, a correlation between the SW propagation speed after MVC and myocardial remodeling in hypertensive heart disease patients was found^10^.

Even though both types of SWE have shown promising results in measuring cardiac stiffness, the differences inherently related to the nature of the SWE techniques used, but also due to variations in experimental settings, complicate a direct comparison of different studies. There are two main inherent characteristics of the SWE methods that can result in differences in SW propagation speed. First, the different time of measurement in both SWE methods with respect to the relaxation/contraction state of the heart affects the measured SW propagation speed^15,23^: natural SWs after valve closure only occur at specific moments during the cardiac cycle, whereas ARF-based SWs can be induced at any time during the cardiac cycle. Second, the temporal and spatial characteristics of natural and actively induced SWs differ due to the differences in the mechanical excitation source. Typically, frequencies up to 500 Hz have been reported for active SWE^28–30^, whereas this is maximally 150 Hz for natural SWE^11,12,19,31^. Therefore, the measured SW propagation speed might be dissimilar due to wave velocity dispersion in the thin-walled myocardium (~ 10 mm). Next to these intrinsic factors, general experimental factors can also cause differences in SW propagation speed, such as the selected echocardiographic view and M-spline location and orientation within the interventricular septum (IVS)^15,32^. For example, for ARF-induced SWs, it is known that measuring their propagation along the myocardial fiber orientation at mid-IVS (as done in a parasternal short-axis view) results in a higher SW propagation speed than measuring across the fiber orientation (as done in a parasternal long-axis view)^27^. Also, natural SWs measured in parasternal or apical view can give various SW propagation speeds due to a difference in the tracked tissue-motion component^33^. Interpretation of cardiac SWE data is thus complex, and it is unsure how these different SW measurements are related to each other and to the mechanical properties of the heart.

The term ‘shear wave elastography’ is generally used in literature^8,9,19,27^ when propagation speeds of transversal wave motion are measured to assess the material’s stiffness. However, it should be noted that the term ‘shear wave’ does probably not describe the complex wave phenomena in the myocardium from a physics point of view. Therefore, from now on, we will use the terms ‘wave’ and ‘wave propagation speed’ throughout this manuscript to refer to the ARF-induced waves and the vibrations after valve closure.

In light of these considerations, the objective of this study is to directly compare the wave speeds in both SWE methods, within individual subjects and heartbeats. A systematic study on the effect of different experimental factors on measured wave propagation speeds as described above is outside the scope of this study. We studied the natural waves after AVC and MVC and compared these in the same echocardiographic view with actively induced waves induced throughout the cardiac cycle in four individual pigs. In this way, the potential effects of differences in experimental factors on wave propagation speeds was minimized. To make a fair comparison between the SWE methods, waves were analyzed in such ways as would have been done when using one individual SWE method. Thus, intrinsic differences in spatial and temporal characteristics of the natural and ARF-induced waves were not circumvented. Until now, inducing and tracking ARF-induced waves throughout the cardiac cycle in a closed-chest transthoracic setting has been challenging^16,34^. Consequently, the present study is performed in open-chest pigs in order to capture the stiffness variations throughout the cardiac cycle with active SWE. In this way, we compared the timing within the cardiac cycle and the spatial and temporal characteristics of the different SWE methods to better understand which aspects of ventricular stiffness are assessed by each method.

## Methods

### Animals

This study included four Yorkshire x landrace female pigs, 5–9 months old and weighing 85.5–100 kg. The study was approved by the Erasmus Medical Center Animal Experiments committee (Nrs. 17-2411-03 for animal 1 and 18-5224-01 for animal 2–4^35^), and all methods were performed in accordance with relevant guidelines and regulations. Mean systolic aortic pressures of 101 mmHg, 77 mmHg and 82 mmHg were measured for pig 2, 3 and 4 respectively. These mean pressures were 76 mmHg, 33 mmHg and 57 mmHg respectively in diastole. No blood pressures were available for the first pig due to absence of such measurement in the research protocol. At time of the experiment, pigs were sedated by an intramuscular injection with a cocktail of Zoletil (tiletamine/zolazepam; 5 mg kg^−1^; Virbac, Barneveld, the Netherlands), Sedazine (xylazine; 2.25 mg kg^−1^; AST farma, Oudewater, the Netherlands) and atropine (2 mg; TEVA, Haarlem, the Netherlands), anesthetized with pentobarbital (9 mg kg^−1^ h^−1^ i.v.; Pharmacy Faculty of Veterinary Medicine Utrecht University, Utrecht, the Netherlands) and mechanically ventilated. Animals were positioned in a supine position to perform a sternotomy. The heart was dissected free without opening the pericardium.

### Data acquisition

SWE measurements were performed with a Vantage 256 ultrasound research system (Verasonics, Kirkland, United States) connected to a P4-2 probe (ATL, Bothell, Washington, United States). The probe was placed inside the animal’s chest with a gel or water stand-off on the right ventricular free wall. Care was taken not to compress the stand-off material with the probe, and thus not to impose an extra pressure on the heart. Live conventional B-mode imaging (frame rate of 50 Hz) was used to find a long-axis parasternal view, with the mid-ventricular part of the IVS wall in the center of the image. The focusing depth of the push beam was then set between mid-IVS depth and endocardial border at the LV side. Live imaging was frozen when a high frame rate (HFR) SWE acquisition was performed, consisting of a natural and an active SWE sequence. Sequences were individually triggered by an analog R-peak output from an ECG module (CWE 3-leads Cardiotachometer CT-1000, Ardmore, United States). Radio frequency (RF) data were saved for offline processing.

A complete HFR SWE acquisition consisted of two consecutive sequences. During the first sequence, the natural waves were imaged for 2 s, using HFR diverging waves (DW) with a minimum frame rate of 2.2 kHz. During the second sequence, waves were induced by repeatedly applying ARF-push pulses during a period of 1.2 s, at a rate of 34 Hz. This resulted in 42 pushes per acquisition. One ARF-based SWE recording consisted of three consecutive stages: (i) HFR DW imaging as pre-push reference (4–20 frames), (ii) focused ARF-push beam transmission with a pulse duration of 400 µs, and (iii) HFR DW imaging to track the wave propagation for 20–28 ms, at a minimum frame rate of 3.5 kHz.

Since different transmission settings have been suggested for SWE in literature^11,18,32,34,36,37^ and since there was no immediate feedback on the success of wave tracking, different settings were considered. We systematically cycled through a predefined scheme of transmission settings during the experiments, for both natural and active SWE. An overview of all data acquisition settings is given in Table 1. The following settings were altered throughout the acquisitions: (i) the virtual diverging-wave focus was either − 34 mm or − 288 mm, in which the far virtual focus theoretically resulted in a higher signal-to-noise ratio (SNR) and lower clutter levels but a reduced field-of-view width to track the wave; (ii) the tracking scheme was either a progressive pulse-inversion scheme (*f*_*0*_ of 2 MHz) or a 3-angle compounding scheme (*f*_*0*_ of 3 MHz); and (iii) the center frequency f_0_ of the ARF-push beam was either 2.0 or 2.8 MHz. Since no superior acquisition scheme was found in terms of wave trackability, all successful SWE measurements (see “Feasibility of a SWE acquisition” section) were retained for further analysis—independent of the selected acquisition settings.

**Table 1:** Overview of the data-acquisition and -analysis settings for the natural and active SWE sequences.

	Natural SWE	Active SWE
**Data acquisition**		
Wave excitation		
Push frequency	–	2.0 MHz or 2.8 MHz
Push duration		400 µs
Wave imaging		
Virtual focus	− 34 mm or − 288 mm	
DW imaging scheme	Progressive pulse-inversion (f_0_ of 2 MHz) or 3-angle compounding (f_0_ of 3 MHz)	
Duration	1.2 s	20 ms or 28 ms, at a rate of 34 Hz during 1.2 s
Frame rate	Minimum of 2.2 kHz	Minimum of 3.5 kHz
RF filter		
Pulse-inversion	Butterworth 10th order BPF 3–4.5 MHz	
Compounding	–	
**Data analysis**		
IQ filter (removal of blood motion and noise)	Butterworth 6th order LPF 250 Hz	–
Tissue motion estimator	One-lag autocorrelation algorithm	
Autocorrelated IQ filter (smoothing)	Gaussian 5.6° by 3.0 mm	Gaussian 1.9° by 1.0 mm
TDI filter (removal of gross motion and noise)	Butterworth 6th order BPF 15–100 Hz	Butterworth 6th order BPF 75–750 Hz
Wave speed estimator	Radon	Manual

SWE: shear wave elastography, RF: radio frequency, BPF: bandpass filter, IQ: in-phase and quadrature, LPF: lowpass filter, and TDI: tissue Doppler imaging.

### Data analysis

Analytic in-phase and quadrature (IQ) data were obtained by offline beamforming the RF data using the Verasonics software (Kirkland, WA). For the pulse-inversion DW images, a 10th order bi-directional Butterworth band-pass filter (3–4.5 MHz) was applied around the second harmonics of the RF data before beamforming. Post-processing of the analytic data was performed in Matlab R2019a (MathWorks, Natick, MA, USA). It is generally known that natural waves have a lower frequency content (maximal 150 Hz^11,12,19,31^ versus 500 Hz^28–30^) and thus larger spatial wavelength than ARF-induced waves. Therefore, different processing settings have been reported for natural and active SWE measurements in literature^11,15,19,29,36,38,39^ (see Supplementary Table S1). To make a fair comparison between the SWE methods, waves were analyzed in such ways as would have been done when using one individual SWE method. An overview of the reasons for applying different processing settings for both methods is given in Supplementary Table S2. Taken into account the settings reported on in literature, the selected data processing settings for both SWE methods in this study are summarized in Table 1. A detailed explanation of the selected data analysis settings for each SWE method is given below. This table shows, for example, that a 3 times larger spatial smoothing filter and a frequency filter with 5–7.5 times lower cut-off frequencies were applied on the natural compared to the ARF-based SWE data, which is consistent with their dominant spatial and temporal wavelengths.

#### Natural SWE

**Figure 1:**
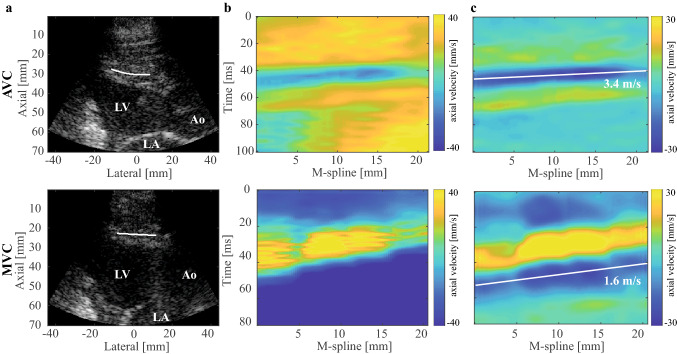
Example of a natural SWE acquisition. (**a**) B-mode at the moment of AVC and MVC, with indication of the considered M-spline. (**b**) M-panels for AVC and MVC depicting the axial tissue velocities as a function of time and space, without band-pass filtering the velocities. (**c**) Same M-panels as in (**b**), now with a 15–100 Hz band-pass filter applied to the tissue velocities. The white line represents the tracked wave by using a Radon transform, with its slope corresponding to the wave speed. LV: left ventricle, LA: left atrium, and Ao: aorta.

To minimize the effect of blood motion and noise, a 6th order bi-directional Butterworth low-pass filter (cut-off frequency of 250 Hz) was applied on the IQ data. Axial particle velocities were obtained by applying a one-lag autocorrelation technique^13^, resulting in tissue Doppler imaging (TDI) data. The one-lag autocorrelation frames were smoothed using a Gaussian spatial smoothing filter of 5.6° by 3.0 mm in the polar domain, before calculating the phase. Anatomic M-mode splines (M-splines) were manually drawn on the basal/mid-ventricular part of the IVS at the moments of AVC and MVC, which were determined from B-mode and corresponding TDI image. The splines followed the curved shape of the IVS, i.e. the assumed propagation direction of the waves. Ten M-splines were drawn in total by two observers at various locations throughout the entire thickness of the IVS where waves were visible (an example for a M-spline is shown in Fig. 1a). Note that different propagation speeds can be obtained for separate M-splines drawn over the IVS in each acquisition, potentially caused by a changing fiber orientation over the thickness, by an inaccurate selection of the propagation direction, and by local variations in tissue stiffness, as previously shown^32^. Imprecision induced by tracking inaccuracies in determining the propagation speeds along individual M-splines was reduced by averaging the propagation speeds obtained for the multiple M-splines drawn by two observers. This way, also the variability caused by M-spline location was included. The axial particle velocities along these M-splines were combined into M-panels, depicting tissue velocity as a function of space and time as shown in Fig. 1b. A 6th-order 15–100 Hz bi-directional Butterworth bandpass filter was applied on the TDI data to remove gross motion. In the M-panels, the waves are shown as wave patterns propagating from base to apex along the M-spline, see Fig. 1b,c. The propagation speeds were obtained by applying a Radon transform on the M-panels^34^, which calculates the integral intensities along all possible linear wave paths in the 2D M-panel and selects the wave path corresponding to the minimum intensity. To prevent any bias, M-panels were first resampled to have an equal number of pixels in space and time, and the Radon domain was normalized^19,39^.

#### Active SWE

**Figure 2:**
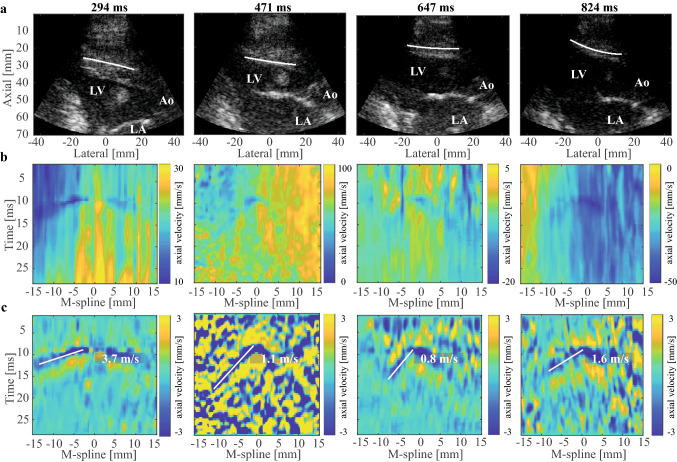
Example of an active SWE acquisition at 4 time points. (**a**) B-mode at 4 different time points across the cardiac cycle. The white spline in this B-mode represents a M-spline. (**b**) M-panels for the considered time points depicting the axial tissue velocities as a function of time and space, without band-pass filtering the velocities. (**c**) Same M-panels as in Fig. 1b, now with a 75–750 Hz band-pass filter applied to the tissue velocities. The white line represents the tracked wave, with its slope corresponding to the wave speed. LV: left ventricle, LA: left atrium, and Ao: aorta.

Similar to the processing of the natural SWE data, a one-lag autocorrelation technique was used to compute the axial particle velocities from the IQ data for active SWE. A Gaussian spatial smoothing filter of 1.9° by 1.0 mm was applied to the autocorrelation frames to reduce the effect of noise. The reverberation frames that occurred after the ARF push were removed (~ 500 µs)^29^. Subsequently, these missing frames between the reference and remaining frames were linearly interpolated to enable down-stream processing. Gross motion and high frequency noise were reduced by applying a 6th-order 75–750 Hz bi-directional Butterworth bandpass filter to the M-panels. Similar as for the natural sequence, 5 M-splines were manually drawn by each observer for every ARF push (see Fig. 2a for example M-splines) to take into account inter-observer and intra-scan variability^39^. The limited SNR of the active M-panels (peak displacement values of ~ 5 µm for active SWE^40^ vs. ~ 100 µm for natural SWE^11,19^) did not permit a robust application of the Radon transform for wave propagation speed estimation, especially in systole where TDI amplitudes are the lowest. Therefore, each observer obtained wave propagation speeds from the slope of a manually drawn line following the negative peak TDI values in a M-panel (see Fig. 2b,c), propagating from the center of the probe towards the apex, as indicated in Fig. 2c. Performing the wave propagation speed analysis by two observers is expected to decrease the subjectivity associated with the manual wave tracking method.

### Feasibility of a SWE acquisition

To obtain representative diastolic and systolic wave propagation speeds without ECG recordings, a large part of the dynamic stiffness variations across the cardiac cycle needed to be captured in order to accurately define the 10% lowest and 10% highest median wave propagation speeds^36^. Therefore, a complete HFR SWE acquisition, consisting of a natural and active SWE sequence, was only included if the ARF-based waves could be tracked for more than 40% of the ARF pushes throughout the cardiac cycle by both observers. This means that SWE recordings with a significant amount of loss of probe contact during the cardiac cycle or with a low SNR for speed estimation were automatically excluded. Additionally, SWE acquisitions where the basal-mid ventricular part of the IVS was not visible, or in which the IVS was not quasi-horizontally oriented (angle with horizontal axis larger than approximately 30°), were also discarded.

### Statistics

Statistical analyses were performed using the statistical toolbox of Matlab. For natural SWE, median values and inter-quartile ranges (IQR) of the wave propagation speeds along 10 M-splines across all heart cycles within one acquisition were computed for AVC and MVC separately. A median wave propagation speed was also calculated for AVC and MVC in each pig individually. For the actively induced waves, the median and IQR of the wave propagation speeds across 10 M-splines were reported for every push in each acquisition. To obtain the median and IQR of the ARF-based wave propagation speed in systole and diastole of one animal, the time traces of individual acquisitions were first temporally matched, as ECG triggering did not properly work for the first two pigs due to signal interference in the operating room. Because ECG signals were not extracted, systolic and diastolic median wave propagation speeds were computed by selecting the 10% highest and 10% lowest median wave propagation speeds for every acquisition respectively^36^. Non-parametric two-sided Wilcoxon signed-rank tests were used to test the significance of measured differences in wave propagation speed. Correlation between wave propagation speeds was determined by computing a linear correlation coefficient (Pearson) and performing linear regression. All *p*-values (two-sided) smaller than 0.05 were interpreted as being significant.

## Results

### Success rate of the SWE acquisitions

**Figure 3:**
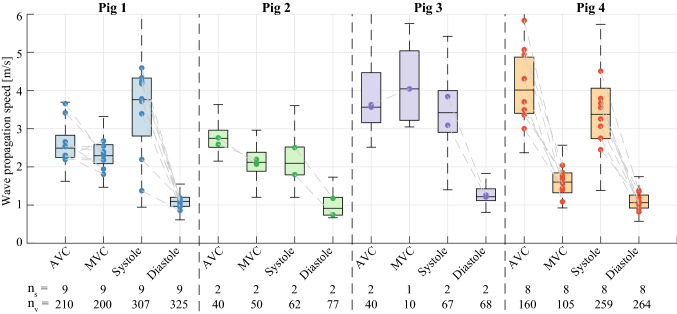
Overview of the natural and ARF-based wave propagation speeds obtained for each pig. The dots depict the median wave propagation speed of all M-splines within one sequence. The boxplots represent the variation of all wave propagation speed values across all M-splines in all sequences. The total number of successful sequences and the total number of wave speed values successfully estimated along M-splines drawn in the successful sequences are indicated by the n_s_- and n_v_-value at the bottom of the boxplot, respectively. Consecutive natural and active SWE sequences are connected by a dotted line. Systolic and diastolic wave speed values were computed over the 10% maximum and 10% minimum median values for each acquisition.

For the ARF-based SWE sequences, 32% (21 out of 65 in total) was considered successful: more than 40% of the waves throughout the cardiac cycle were tracked in these sequences. The remaining active and corresponding natural SWE data were excluded from our analysis. Figure 3 gives an overview of the successfully estimated propagation speeds for natural and ARF-based SWE in each pig individually. The dots in the figure depict the median values per successful sequence and the total number of successful sequences per animal is given by the n_s_-value stated at the bottom of the figure. For all recorded ARF-based sequences within each pig, success rates of 36% (9/25), 14% (2/14), 13% (2/15) and 73% (8/11) were obtained respectively. Within the corresponding natural sequences, wave propagation speeds after AVC and MVC were successfully determined in 100% (21/21) and 95% (20/21) of the sequences, respectively.

The boxplots in Fig. 3 represent the wave propagation speed variations across all M-splines in the successful acquisitions of the individual pigs, in which the number of successful wave propagation speed values is indicated by the n_v_-value mentioned at the bottom of the figure. For ARF-based SWE, diastolic wave propagation speeds were successfully determined for 90% (325/360), 96% (77/80), 85% (68/80) and 83% (264/320) of all considered M-splines for pigs 1 to 4, whereas the success rate was slightly lower in systole: 85% (307/360), 78% (62/80), 84% (67/80) and 81% (259/320). Nonetheless, median diastolic and systolic propagation speed values were obtained for all 21 successful ARF-based sequences, as indicated by the n_s_-values in Fig. 3. For natural wave propagation speed estimations, the total number of AVC and MVC events varied from 1 to 3 per sequence, depending on the heart rate of the pig. Wave propagation speeds after AVC were successfully determined in 100% (210/210), 100% (40/40), 100% (40/40) and 100% (160/160) of all M-splines for each pig. For MVC, successful wave propagation speed estimations were obtained for 100% (200/200), 100% (50/50), 33% (10/30) and 81% (105/130) of the M-splines analyzed for each pig individually. It should be stressed, that although the success rate of determining propagation speeds corresponding to individual M-splines drawn was lower for MVC than for AVC, still in 20 of the 21 sequences, median MVC propagation speed values could be obtained, as indicated by the n_s_-values in Fig. 3.

### Natural SWE

An overview of the natural wave propagation speeds for each pig individually is illustrated in Fig. 3; with the number of successful sequences n_s_ being equal to 9, 2, 2 and 8 for AVC, and 9, 2, 1 and 8 for MVC, in animal 1–4 respectively. For AVC, median propagation speeds of 2.5 m/s (IQR: 2.2–2.8 m/s), 2.7 m/s (IQR: 2.5–3.0 m/s), 3.6 m/s (IQR: 3.2–4.5 m/s) and 4.0 m/s (IQR: 3.4–4.9 m/s) were obtained for the pigs individually. For MVC, these values are 2.3 m/s (IQR: 2.1–2.6 m/s), 2.1 m/s (IQR: 1.9–2.4 m/s), 4.0 m/s (IQR: 3.2–5.0 m/s) and 1.6 m/s (IQR: 1.3–1.8 m/s) for each pig respectively. The median propagation speeds after AVC were found to be significantly higher than after MVC for the total set of sequences among all four animals (Wilcoxon signed-rank test, p < 0.01, n_s_ = 20). The ratios of the median propagation speed after AVC and MVC for each individual pig are 1.1, 1.3, 0.9 and 2.5. No statistical differences were found between the median natural wave propagation speeds obtained by the two observers (Wilcoxon signed-rank test, p = 0.46, n_s_ = 41), and a very strong linear correlation was found (Pearson, r = 0.90, p < 0.01).

### Active SWE

**Figure 4:**
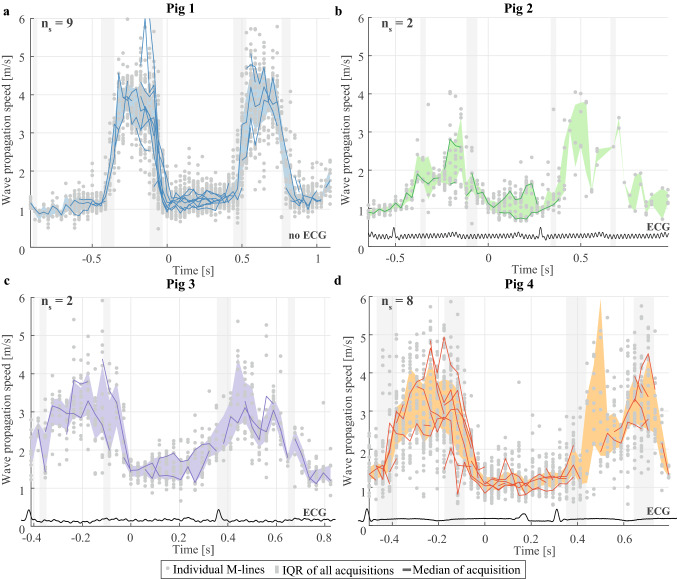
Overview of the propagation speeds obtained for the active SWE sequence. Individual sequences were aligned in time for each pig individually (**a**–**d** respectively), with the onset of diastole set to t = 0 s. The dots depict the wave propagation speed values obtained with individual M-splines, while the solid lines show the median wave speeds of all M-splines within one sequence (if n_v_ ≥ 5). The shaded colored areas depict the inter-quartile range of all wave speed values for all active sequences per animal. The grey shaded vertical bands depict the temporal ranges in which valve closure was observed. The total number of successful active SWE sequences is indicated by the n_s_-value at the top of the figures. Representative ECG signals are also shown in this figure, but were not recorded simultaneously with the SWE acquisitions. For pig 1, no ECG data was saved due to mal-functioning of the ECG device. Note additionally that ECG triggering of the SWE acquisitions did not properly work for pig 2.

Figure 4 shows the propagation speeds obtained throughout the cardiac cycle for all ARF-based sequences per pig. Depending on the heart rate of the animal, one or more heart cycles were captured in the individual sequences of 1.2 s. The solid lines depict the median propagation speed values obtained for the M-splines drawn per ARF-based SWE measurement (number of successful sequences n_s_ is 9, 2, 2, and 8 for pigs 1–4 respectively). After selecting the 10% highest and 10% lowest median wave propagation speeds in each active SWE sequence (corresponding to the wave speed estimations of four pushes within one active SWE sequence), median systolic and diastolic values were computed per active SWE sequence, as shown in Fig. 3. The median systolic wave propagation speeds for the selected M-splines in all acquisitions were found to be 3.8 m/s (IQR: 2.8–4.3 m/s), 2.1 m/s (IQR: 1.8–2.5 m/s), 3.4 m/s (IQR: 2.9–4.0 m/s) and 3.4 m/s (IQR: 2.7–4.1 m/s) for each pig individually. The median diastolic wave propagation speeds are 1.1 m/s (IQR: 1.0–1.2 m/s), 0.9 m/s (IQR: 0.7–1.2 m/s), 1.2 m/s (IQR: 1.1–1.4 m/s) and 1.1 m/s (IQR: 0.9–1.3 m/s) for each pig respectively. The median propagation speeds in systole were found to be significantly higher than in diastole for all acquisitions (Wilcoxon signed-rank test, p < 0.01, n_s_ = 21). The ratios of the median propagation speed in systole and diastole for each individual pig are 3.4, 2.3, 2.8 and 3.2. No statistical differences were found between the median systolic and diastolic wave propagation speeds obtained by the two observers (Wilcoxon signed-rank test, p = 0.71, n_s_ = 42), and a very strong linear correlation was found (Pearson, r = 0.93, p < 0.01).

### Natural versus active SWE

**Figure 5:**
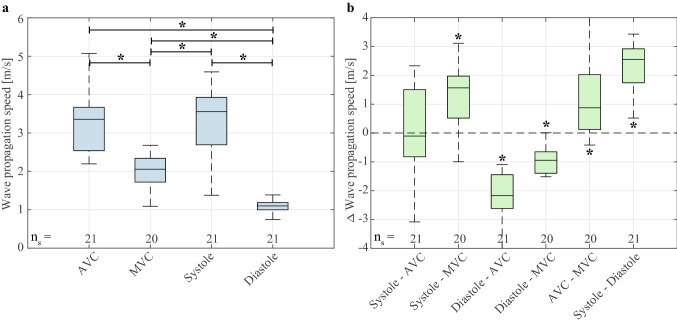
(**a**) Boxplots of the median wave propagation speed values obtained per acquisition for the natural and ARF-based waves. (**b**) The propagation speeds of the different types of waves are compared by matching the values obtained in individual sequences (for AVC vs. MVC and for systole vs. diastole) or in acquisitions (for natural vs. active SWE in consecutive heart beats). Asterisks (*) denote a significance level of p < 0.05 for a two-sided Wilcoxon signed-rank test. All propagation speeds were found to be significant different, except from the natural waves after AVC and the ARF-based waves in systole.

The median propagation speeds for the individual natural sequences are compared with the corresponding median values in systole and diastole in Fig. 5. The propagation speeds after MVC were found to be higher than the wave speeds in diastole (Wilcoxon signed-rank test, p < 0.01, n_s_ = 20), and lower than in systole (Wilcoxon signed-rank test, p < 0.01, n_s_ = 20). The wave propagation speeds after AVC were also found to be higher than in diastole (Wilcoxon signed-rank test, p < 0.01, n_s_ = 21), but no significant difference was found compared to systole (Wilcoxon signed-rank test, p = 0.99, n_s_ = 21). Note that especially for pig 4 propagation speeds after AVC are higher than wave propagation speeds in systole, while for the other pigs most wave propagation speed values after AVC are comparable or below systolic speeds.

### Natural waves in active SWE

In most active SWE sequences, natural waves induced by AVC and MVC were also visible. To determine their propagation speeds, the same data-analysis methods were used as previously described for natural SWE. For AVC, we were able to estimate its wave propagation speed in 44% (4/9), 100% (2/2), 50% (1/2) and 100% (8/8) of the active SWE sequences for each pig, respectively. Wave speed estimation after MVC was successful in 67% (6/9), 100% (2/2), 50% (1/2) and 100% (8/8) of the active SWE sequences for each pig, respectively. For AVC, median propagation speeds of 2.5 m/s (IQR: 2.0–2.7 m/s), 2.8 m/s (IQR: 2.4–3.7 m/s), 2.0 m/s (IQR: 1.4–2.1 m/s) and 3.4 m/s (IQR: 2.5–4.1 m/s) were obtained for pigs 1 to 4 respectively. For MVC, these values are 2.1 m/s (IQR: 1.9–2.6 m/s), 2.4 m/s (IQR: 2.2–2.5 m/s), 3.4 m/s (IQR: 3.0–3.9 m/s) and 2.0 m/s (IQR: 1.6–3.1 m/s) for each pig. Despite the ARF-based waves being simultaneously present, no significant difference was found between the natural wave propagation speeds in the active SWE sequences and the ones in the natural SWE sequences for AVC (Wilcoxon signed-rank test, p = 0.23, n_s_ = 15) and MVC (Wilcoxon signed-rank test, p = 0.49, n_s_ = 17).

**Figure 6:**
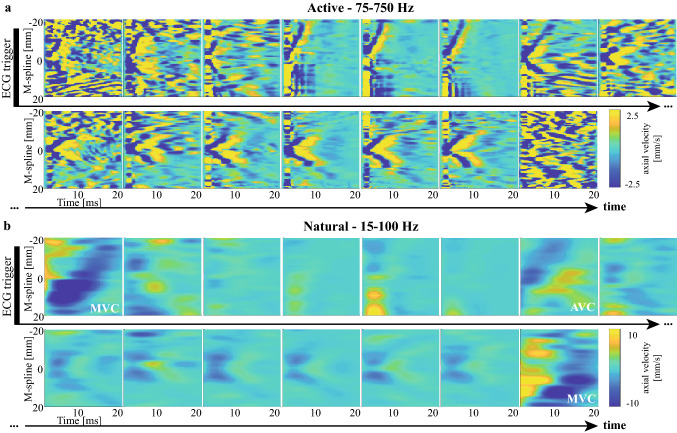
Effect of filter settings on wave visibility in M-panels of an active SWE sequence. (**a**) Various M-panels are displayed for different ARF pushes throughout the cardiac cycle, after applying a 75–750 Hz bandpass filter to the TDI data. (**b**) The same M-panels as in (**a**) are shown after applying a 15–100 Hz bandpass filter to the TDI Data, as done for natural SWE processing. Events of mitral and aortic valve closure (AVC and MVC) are denoted in their respective frames.

**Figure 7:**
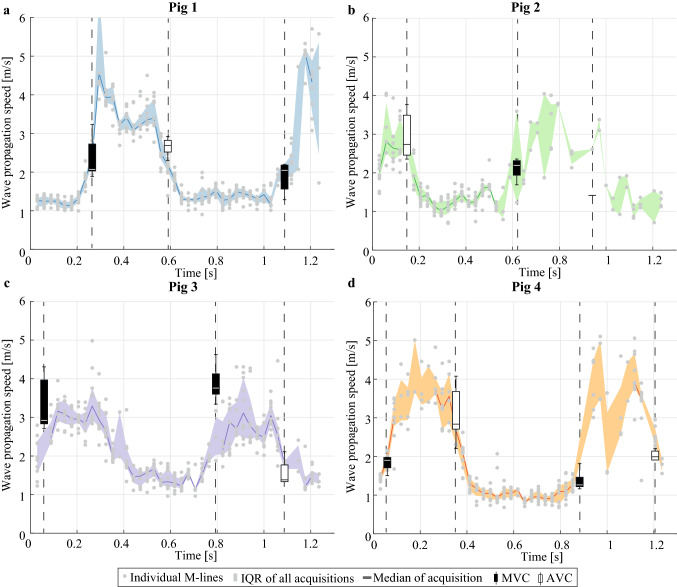
Example of an active SWE sequence for each individual pig, in which the natural waves after AVC and MVC were tracked for the same M-spline in the same sequence. The dots depict the wave speed values for individual M-splines, while the solid lines and shaded areas show the median values and inter-quartile ranges, respectively. Note that no data is shown for the first AVC event in pig 3 as wave tracking after AVC was not feasible for this specific sequence due to the limited temporal recording length.

**Figure 8:**
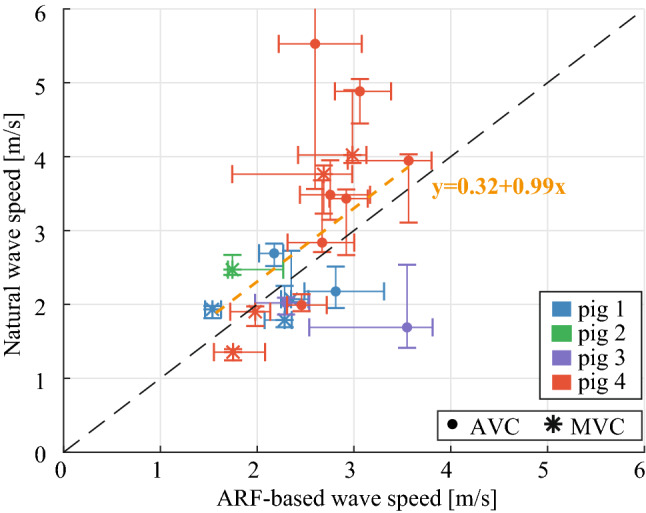
Comparison of the median propagation speed values of the natural waves within the active sequences, and the active median propagation speeds measured at the same time in the cardiac cycle as the corresponding natural wave. The orange dotted line depicts the linear regression fit of the data.

The spatial and temporal characteristics of natural and actively induced waves are compared in Fig. 6, visualizing M-panels obtained for an individual active SWE sequences after applying the active SWE filter settings (panel A), and after applying the natural SWE filter settings (panel B). Note that the same M-splines were used for the M-panels shown in Fig. 6a,b. A larger period (~ 15 ms vs. ~ 2 ms), spatial wavelength (a few cm vs. a few mm) and TDI amplitude (~ 10 mm/s vs. ~ 2.5 mm/s) was found for the natural waves than for the ARF-based waves. Figure 7 shows the wave propagation speed estimations of examples of active SWE sequences in each pig together with the natural wave propagation speed. Tracking natural waves within active SWE sequences allows to compare natural and ARF-based wave propagation speed values at the same moment within the cardiac heart cycle, as shown in Fig. 8. No statistical differences were found between the natural and ARF-based wave propagation speeds (Wilcoxon signed-rank test; AVC: p = 0.41, n_s_ = 11, MVC: p = 0.46, n_s_ = 8). Even though only a weak linear correlation was found between the natural and ARF-based wave propagation speeds (Pearson, r = 0.48, p = 0.04), the slope of the corresponding linear regression was found to be 0.99 (R^2^ = 0.23), indicating a one-to-one relationship between natural and ARF-based wave propagation speeds (see also Fig. 8). However, note that in this figure some propagation speeds after AVC for pig 4 are higher than the corresponding ARF-based wave speed.

## Discussion

Natural and active shear wave elastography (SWE) are promising non-invasive methods to potentially improve the current assessment of the mechanical properties of the heart. However, the relationship between both techniques, and which aspect of cardiac ventricular stiffness is assessed by each method remained unclear. Therefore, in this study we directly compared natural waves after aortic and mitral valve closure (AVC and MVC) with waves induced throughout the entire cardiac cycle using an acoustic radiation force (ARF), in four open-chest pigs. The results show that natural wave propagation speeds match well with the ARF-based wave speeds measured at the moment of valve closure, despite the differences in temporal and spatial characteristics of the waves. Additionally, analysis of the natural waves in the active SWE acquisitions showed that natural waves occur in between the diastolic and systolic phases identified by ARF-based wave propagation speeds, and have higher propagation speeds than in diastole. Therefore, natural wave propagation speeds not only relate to the myocardial stiffness in relaxation but also to the contractile state.

### Natural versus actively induced wave characteristics

The excitation sources of natural and actively induced waves have different characteristics, which affect the wave properties. First, the orientation of the excitation force differs: active SWE emits a force mainly in the direction of the ultrasound beam (i.e. transversal with respect to the IVS in parasternal view)^41^, whereas the exact orientation of the force for natural SWE is unknown. However, the force after AVC will probably not be purely transversally oriented because both a transversal (parasternal view) as well as a longitudinal component (apical view) of tissue motion in the IVS has been measured in an earlier study^33^. Second, the temporal and spatial characteristics of the excitation source are completely different, which might affect the spectral content of the wave^42^. In active SWE, the ARF’s spatial and temporal characteristics are theoretically determined by the push settings (push duration, center frequency, etc.) and the acoustic properties of the medium^43^. Ideally, the ARF acts as a volume force with a focal extent throughout the entire IVS thickness in order to generate a wave propagating over the complete IVS. In natural SWE, the exact temporal and spatial characteristics of the wave source are unknown, but are determined by a complex interaction between the leaflet dynamics, blood pressure changes and local (back)flow dynamics. Natural waves are excited by a local force on the IVS near the valve and gradually propagate from this location to the entire IVS thickness.

Due to the different excitation sources, the natural and actively induced wave characteristics vary in terms of period, wavelength and velocity magnitude, as visualized in Fig. 6. As natural waves have a lower frequency content than actively induced waves, earlier literature reports different TDI filter settings^11,15,19,29,36,38,39^ depending on the wave analyzed (Supplementary Table S1). To make a fair comparison between the SWE methods, waves were analyzed in such ways as would have been done when studying only one SWE method. Therefore, the current filter settings of 15–100 Hz vs. 75–750 Hz were selected based on these literature reports and the observed frequency content in our SWE data. Indeed, Fourier analysis of TDI-panels of both natural and ARF-based waves showed that by applying the different filters for the different SWE methods, the relevant wave content was preserved (see Supplementary Figure S1). The discrepancy around the lower cut-off frequency between the Fourier spectrum of the unfiltered and filtered ARF-based wave was needed to separate the wave from low-frequency general heart motion with relatively large amplitudes, often complicating the tracking process of these waves as shown in Fig. 2. Supplementary Figure S1 shows an approximated 25 dB lower signal at the upper cut-off frequency, which validates our choice of the upper cut-off frequencies. Furthermore, despite the ARF-based waves being simultaneously present, no significant difference was found between the natural wave speeds within the active SWE sequences and within the natural SWE sequences. This suggests that natural waves could be correctly separated from the ARF-induced waves, and that the ARF-induced waves that were simultaneously present with the natural waves did not cause a bias in the natural propagation speeds obtained. Assuming that antisymmetric zero-order Lamb wave modes were induced, the lower frequency content would make natural waves theoretically more sensitive to wave dispersion^12^, potentially decreasing the wave propagation speed measured. However, the smaller bandwidth of natural waves compared to actively induced waves limits frequency dispersion and consequent wave distortion. Furthermore, Urban et al.^44^ showed using finite element simulations of impulsive and harmonic excitations that low-frequency waves are less sensitive to fiber orientation than high-frequency waves, resulting in less wave guiding along the fiber. The changing myocardial fiber orientation across cardiac thickness^45^ would thus theoretically have less impact on the natural waves, resulting in less wave speed variation across the IVS thickness when neglecting other relevant factors.

This study shows higher wave propagation speeds for the waves after AVC and MVC (median speed range of 2.2–5.8 m/s and of 1.1–4.0 m/s respectively) than for the actively induced waves in diastole (median speeds between 0.7–1.4 m/s), as demonstrated in Fig. 5. The ARF-based wave propagation speeds in systole (median range of 1.4–4.6 m/s) were found to be higher than the wave propagation speeds after MVC (median range of 1.1–4.0 m/s), but not statistically different from the wave speeds after AVC (median range of 2.2–5.8 m/s). Besides the different wave characteristics, it should be noted that—since natural and active SWE sequences were performed in consecutive heartbeats for Figs. 3 and 5—part of the wave propagation speed differences could be caused by M-spline location, cardiac natural variations (inter-beat variability), and the moment within the cardiac cycle^15,39^. However, the second analysis, where we measured the natural and actively-induced waves simultaneously for the same M-splines, a good correspondence between natural and ARF-based wave propagation speed was found in Figs. 7 and 8. This indicates that they provide equal information about the cardiac stiffness at that time instance, despite their differences in wave characteristics as described above.

The reported median wave speed values per animal for natural SWE (median of 1.6–4.0 m/s for MVC and 2.5–4.0 m/s for AVC) and active SWE (median of 0.9–1.2 m/s in diastole and 2.1–3.8 m/s in systole) are within the ranges documented in literature for the IVS in other porcine studies. For natural SWE, Vos et al.^19^ obtained a median propagation speed of 2.2 m/s after MVC (90% interval: 0.8–3.2 m/s) and 4.2 m/s after AVC (90% interval: 1.4–6.3 m/s) in 22 closed-chest pigs. For active SWE, Hollender et al.^16^ reported a range of median velocities between 0.89 and 2.20 m/s in diastole and 2.60 and 5.14 m/s in systole for 6 closed-chest pigs using an intracardiac probe. Although similar range of values have been reported in other animal species and even in human subjects, other species might have inherently different cardiac properties, and therefore we refrain from comparing our wave speed results with non-porcine studies.

### Timing of natural waves within the heart cycle

Valve closure is commonly used to divide a cardiac cycle into a diastolic and systolic phase, in which AVC typically describes the end of systole or the beginning of diastole and MVC the beginning of systole or the end of diastole^46^. However, the exact timing of the valve closure events with respect to the dynamic stiffness variation of the heart is unknown, and therefore it is uncertain in what relaxation and/or contraction state of the heart the natural wave measurements are performed. We measured higher wave propagation speeds after AVC than after MVC, as reported on by several other studies^9,11,19,39^, suggesting a higher myocardial stiffness at the moment of AVC than of MVC. Figure 7 compares natural and actively induced waves within the same heartbeat, substantiating our expectation that wave tracking after MVC occurs during the period in between diastolic and systolic speeds and wave tracking after AVC during the period in between systolic and diastolic speeds. Due to the timing of natural waves, both AVC and MVC measurements are representative for an instantaneous myocardial stiffness that does not only depend on the passive cardiac material properties but also on the loading condition and the active relaxation/contractility characteristics. This is also supported by the higher wave propagation speeds measured for natural SWE than for active SWE in diastole.

Although this study confirms that the waves after AVC and MVC occur during the phases that the heart changes contractile state (as assessed with active SWE), the exact moments within these phases could vary between test subjects and even between heartbeats. Valve closure is dictated by the pressure differences between left ventricle and atrium or aorta, and a time delay might be present between the moment of valve closure and the recording of the wave further down the septal wall along the selected M-spline^31^. For example, for pig 4 in Fig. 7d, lower median natural propagation speeds were measured in the second heartbeat (1.3 m/s for MVC and 2.0 m/s for AVC) than in the first (1.9 m/s for MVC and 2.8 m/s for AVC). This inter-beat variability is in line with the inter-scan variability previously reported in healthy volunteers^39^: IQR of 0.26–0.94 m/s for MVC and 0.40–0.86 m/s for AVC. Furthermore, in contrast to the general findings of this study, the specific sequences in Fig. 7b,c (pigs 2 and 3) show lower wave propagation speed after AVC than after MVC (also visible in Fig. 3 for pig 3). A lower propagation speed for AVC was also found by Strachinaru et al.^8^ in 45 healthy volunteers (mean of 3.51 m/s for AVC vs. 4.68 m/s for MVC). Both observations might be related to a timing difference of valve closure between heartbeats, subjects or even species, but also to measurement inaccuracies (in wave speed estimation, echocardiographic view, and M-spline location) or to changes in the global cardiac dynamics affecting the pressure difference across the valve. These variabilities demonstrate the importance of recording multiple heartbeats to improve the accuracy^39^.

### Clinical interpretation and future work

The objective of this work is to highlight the essential differences in wave characteristics (i.e. speed, wavelength, and wave magnitude) measured with natural and active SWE, and to discuss their clinical interpretation. Both SWE methods have yet demonstrated potential added value in various clinical cardiac studies^8–10,27^, however it was unsure whether the techniques assess a similar stiffness property of the heart. Systolic wave propagation speeds as well as wave speeds after AVC have both been proposed to be potentially related to myocardial contractility^23,47^. No statistical difference was found between the wave propagation speeds after AVC and in systole in our study. However, it should be noted that the propagation speeds after AVC (3.0–5.8 m/s) seem higher than in systole (2.5–4.5 m/s) for pig 4 in Fig. 3. Furthermore, while pig 1 and 4 show similar systolic wave propagation speed (around 3.6 m/s in Fig. 3), the wave speeds after AVC are different (2.5 m/s in pig 1 vs. 4.0 m/s in pig 4 in Fig. 3). Although other studies^23,47^ showed a correlation with contractility for the propagation speeds in systole and AVC separately, the results of this study suggest that their speed magnitude is not necessarily equal. This could be caused by the different wave characteristics, the timing of the waves after AVC with respect to the functional state of the heart (as suggested by the location of the grey areas with respect to the systolic speeds shown in Fig. 4) and measurement inaccuracies. The small sample size of this study prevents making definite claims on the link between systolic wave speed and wave speed after AVC. In literature, wave speeds in diastole are regarded as a measure for passive myocardial stiffness^17,27^. The ARF-based wave propagation speed values in diastole were very similar for all tested subjects in this study (around 1.1 m/s in Fig. 3). Furthermore, the variability of diastolic wave propagation speeds is much lower than for other reported natural and active metrics, which is advantageous for clinical diagnosis. On the contrary, it is unclear what the waves caused by MVC clinically represent as they do not strictly measure myocardial properties in end-diastole. Whether all findings of this study can be extrapolated to humans, needs to be investigated in future large-scale studies which apply both natural and active SWE. Clinical relevance of both natural and active SWE should be further demonstrated in the future by extending the current study with the assessment of cardiac function and stiffness via pressure–volume loops.

Both natural and active SWE have their specific advantages that might be beneficial for clinical use. Natural SWE can be easier implemented in clinical practice as no high-energy ARF pushes are needed to induce waves, setting less demanding power requirements on the scanner and posing less issues concerning acoustic safety. Furthermore, until now, the feasibility of natural SWE is in general higher than for active SWE, due to higher SNR and the absence of limiting factors for the wave excitation source to reach the IVS while meeting safety criteria (especially challenging in obese patients^34^). On the other hand, applying active SWE gives the user theoretically the freedom to control the spatial and temporal characteristics of the excitation source at any time point in the cardiac cycle. The smaller spatial wavelengths of ARF-based waves compared to natural waves (a few mm vs. a few cm) also results in more accurate ARF-based wave tracking within the limited spatial domain of the selected field-of-view (typically 3–4 cm). Additionally, the wave is always tracked in line with the excitation source for active SWE, whereas out-of-plane motion in natural SWE can cause wave speed estimation errors^19^. However, our study showed a low feasibility rate of 32% for ARF-based SWE in an open-chest setting. Within these successful acquisitions, wave tracking occurred successfully in 89%, 82%, 100% and 79% of all M-splines on average for diastolic waves, systolic waves, waves after AVC and waves after MVC, respectively. Success rate of wave speed estimation after MVC is explicitly lower than that for waves after AVC, as previously reported^39^. In closed-chest settings, to the best of our knowledge, there are no feasibility studies available for ARF-based SWE throughout the cardiac cycle. However, a feasibility rate of 41% throughout the cardiac cycle has been reported in healthy volunteers for another ARF-based method called ARF imaging (ARFI)^48^—using focused pulses to track the on-axis displacement relaxation after ARF application instead of DWs to image the off-axis displacement propagation patterns as in SWE. Due to this difference in displacement tracking, ARFI has typically higher tracking SNR than SWE, explaining the higher feasibility rate. Finding a work-around for this technological limitation in ARF-based SWE remains thus an important task for the future.

### Study limitations

The feasibility rate of active SWE in this study was low: only 32% of all active SWE sequences fulfilled our predefined metric of success (wave visibility in more than 40% of the pushes throughout the cardiac cycle). This resulted in low sample sizes for all statistical tests. Especially the systolic phase set challenges to wave tracking due to a stiffer myocardium resulting in a decreased SNR and therefore a limited spatial window to track higher wave propagation speeds (typically around 15 mm as can be seen in Figs. 2 and 6). The open-chest nature of our experiments is also partly responsible for these low success rates, even though it gave access to the dynamic changes in wave speed across the cardiac cycle. It was difficult to keep the probe in a stable position within an open thorax on a beating heart without losing probe contact, while keeping the IVS in an approximately horizontal position. Depending on the position of the heart with respect to the opening of the thorax and the ribs, this was more difficult for some pigs than for others, leading to a wide variety in success rate of the ARF acquisitions among the pigs. The low SNR for active SWE did also not permit implementation of an automatic method such as the Radon transform to estimate wave speeds. Instead, a manual method was used to get a robust estimate of wave speed, but this method is time-consuming and potentially observer dependent. Wave visibility thus played a role in wave speed estimation and therefore the chosen inclusion criterion for a complete HFR SWE acquisition was also based on wave visibility throughout the cardiac cycle by 2 observers, which is partly subjective. Technical innovations are however still ongoing to further improve SNR for ARF-based SWE measurements, such as dedicated probe development for transthoracic acquisitions or efforts for elevating acoustic output in SWE^49^. These improvements are needed for a more robust and universal method of wave tracking and a quantitative assessment of data quality for objective acquisition inclusion criteria, as well as for increasing the feasibility of transthoracic measurements. During the experiment, no ECG signal was recorded and therefore wave speed in diastole and systole were determined based on the 10% minimal and 10% maximal median wave speed values^36^. Furthermore, since no ECG signal was saved, the timing of natural waves was only compared with the speed dynamics measured with active SWE.

Due to limitations in the data acquisition system, the actively induced waves could not be tracked continuously, but were saved in blocks of 20 or 28 ms after each individual ARF push. Although the saved temporal window was large enough for actively induced wave tracking, it was more challenging to track the natural waves in these active sequences due to their longer wave period. Therefore, natural wave speeds were tracked more reliably in the individual natural SWE sequences with continuous tracking (resulting in a larger temporal window for tracking), although no statistical differences were found with the natural wave speeds in the active sequences. As a result, to make a fair comparison with optimal processing conditions for the different methods, Figs. 3 and 5 show the results for natural and actively induced waves in consecutive heartbeats. The natural waves in active sequences were used to include the effect of timing of these natural waves in Figs. 7 and 8.

## Conclusion

This work studied the relationship in four open-chest pigs between waves naturally occurring after aortic and mitral valve closure, and those actively induced throughout the entire cardiac cycle using an acoustic radiation force (ARF), to bridge the knowledge gap between two groups of reported shear wave elastography (SWE) studies. Despite the shorter wavelength and period of the actively induced waves, a good match in propagation speed was found for both natural and active SWE when actively induced waves were measured at the moment of valve closure. In addition, our simultaneous recordings showed that the waves after valve closures occur in between the diastolic and systolic phases identified with active SWE, and have higher propagation speeds than in diastole. This confirms that natural SWE measures a mixed state of relaxation and contraction, which might hamper easy interpretation in clinical diagnosis for diastolic and/or systolic function. On the other hand, despite the easy timing of stiffness measurements in active SWE, its feasibility was lower than natural SWE, especially in systole. Clinical validation is further needed to exploit both techniques for the assessment of diastolic and systolic function.

## Supplementary information

Supplementary Information.

## Data Availability

The datasets analyzed during the current study are available from the corresponding authors on reasonable request.
