# Supplementary material for: A direct comparison of natural and acoustic-radiation-force-induced cardiac mechanical waves
Source: Sci Rep. 2020 Oct 28;10:18431. doi: 10.1038/s41598-020-75401-1 (PMC7595170; doi:10.1038/s41598-020-75401-1)
Supplement: Supplementary file 1 — Supplementary Information. [file 41598_2020_75401_MOESM1_ESM.pdf]

A Direct Comparison of Natural and Acoustic-Radiation-Force-Induced Cardiac Waves

Lana B.H. Keijzer[1, #, *], Annette Caenen[1,2,3, #, *],
Jason Voorneveld[1], Mihai Strachinaru[1], Daniel J. Bowen[1], Jens van de Wouw[1], Oana Sorop[1], Daphne Merkus[1], Dirk J. Duncker[1], Antonius F.W. van der Steen[1,4], Nico de Jong[1,4], Johan G. Bosch[1], Hendrik J. Vos [1,4]

[#]Both authors contributed equally
*Corresponding authors: l.b.h.keijzer@erasmusmc.nl, annette.caenen@ugent.be

[1]Department of Cardiology, Erasmus MC, Rotterdam, the Netherlands
[2]IBiTech-bioMMeda, Ghent University, Ghent, Belgium
[3]Cardiovascular Imaging and Dynamics Lab, Catholic University of Leuven, Leuven, Belgium
[4]Department of Imaging Physics, Delft University of Technology, Delft, the Netherlands

---

Supplementary Figures

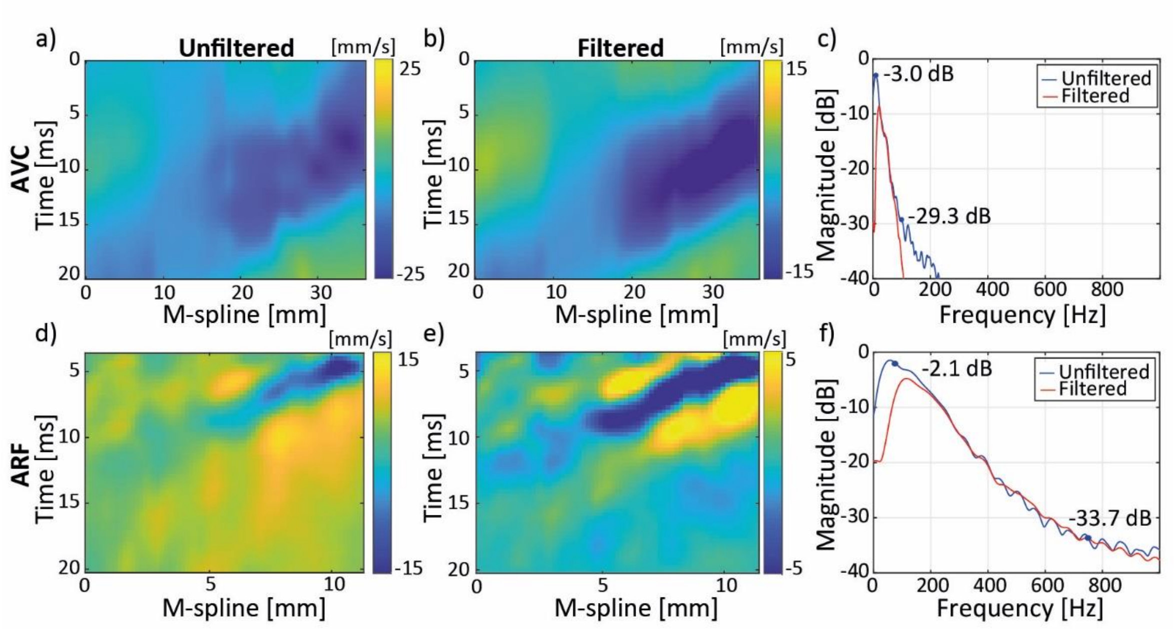

Figure S1: Examples of M-panels obtained for a wave after AVC **(a)** and for an externally induced wave using an ARF **(d)**. Bandpass filters (6th-order Butterworth) of 15 **–** 100Hz and 75 **–** 750Hz were applied on the natural **(b)** and active **(e)** M-panels respectively. The Fourier spectra of the unfiltered and filtered M-panels are shown in **(c)** and **(f)**. Magnitude values at the cut-off frequencies are shown.

Supplementary Tables

Table S1: Overview of reported processing settings in literature (this overview does not represent a complete overview of all SWE studies). Filter settings that show similarities as the settings used in our study are shown in red. Abbreviations: RF: radio frequency, IQ: in-phase and quadrature, DWI: diverging wave imaging; PIHI: pulse-inversion harmonic imaging, NR: not reported, SWE: shear wave elastography, BPF: bandpass filter, HPF: highpass filter, CFWI: clutter filter wave imaging, and LPF: lowpass filter.

		**SWE imaging**	**RF filter**	**IQ filter**	**Wave motion**	**Spatial smoothing**	**Motion filter**
**Active SWE**	Couade *et al.*, IEEE TMI, 2011	DWI in open-chest sheep hearts	NR	NR	Velocity data	NR	Average wall motion is subtracted
	Vejdani *et al.*, IEEE TMI, 2015	DWI in Langendorff-perfused rabit hearts	Data with correlation coefficient < 0.9 were removed	NR	Velocity data	NR	Quadratic motion filter
	Hollender *et al.*, IUS, 2017	PIHI in *in vivo* human hearts	NR	NR	Velocity data	NR	Butterworth 3rd order BPF (75 – 750 Hz)
	Deng *et al.*, IEEE TUFFC, 2017	DWI: general guidelines	NR	NR	Displacement data	Axial averaging over depth of field of push	BPF
**Natural SWE**	Vos *et al.*, UMB, 2017	DWI in closed-chest pig hearts	NR	NR	Velocity data	2.5 x 2.5 mm	Butterworth BPF (15 – 100 Hz)
	Santos *et al.*, IEEE TUFFC, 2019	Multi-zone DWI in *in vivo* human hearts	Gaussian BPF (3.5 MHz, 60% bandwidth)	NR	Acceleration data (=high-pass motion filter)	2 mm x 2.4º	Moving average of 3 frames
	Keijzer *et al.*, UMB, 2019	PIHI in *in vivo* human hearts	NR	Butterworth LPF (250 Hz)	Velocity data	4 mm x 6.7º	Butterworth 6th order BPF (15 – 100 Hz)

Table S2: Overview of the reasons for applying different processing settings on the natural and active SWE sequences. Filter settings were based on settings described in literature (see Supplementary Table S1). Abbreviations: IQ: in-phase and quadrature, LPF: lowpass filter, SWE: shear wave elastography, and ARF: acoustic radiation force.

**IQ Filter**	An LPF with a cut-off value of 250 Hz was applied on the natural SWE sequence to remove data corresponding to blood motion and noise. Since ARF-induced waves have a higher frequency content, applying such filter would go hand in hand with removal of the wave motion data.
**Spatial smoothing**	Natural waves have larger wavelengths (as a consequence of the low frequency spectrum) than ARF-induced waves, and therefore the Gaussian smoothing filter applied on the one-lag autocorrelation frames was larger (5.6º by 3.0 mm versus 1.9º by 1.0 mm).
**Motion filter**	Bandpass filters were chosen such that frequency content of the specific wave type remained, while increasing the robustness of determining wave propagation speeds by filtering out low-frequency gross motion and high-frequency jitter.
